# Heuristic Optimization of a New Type of Prestressed Arched Truss

**DOI:** 10.3390/ma15228144

**Published:** 2022-11-17

**Authors:** Gaioz Partskhaladze, Julian Alcala, Elguja Medzmariashvili, Gocha Chavleshvili, Bichiko Surguladze, Víctor Yepes

**Affiliations:** 1 Engineering and Construction Department, Faculty of Technologies, Batumi Shota Rustaveli State University, 35/32, Ninoshvili/Rustaveli Str., 6010 Batumi, Georgia; 2 ICITECH, Universitat Politècnica de València, Camino de Vera, s/n, 46071 Valencia, Spain; 3 Department of Civil and Industrial Engineering, Georgian Technical University, 77 Kostava Str., 0175 Tbilisi, Georgia

**Keywords:** prestressed truss, stiffness matrix method, tensile element, compressed element, optimization, simulated annealing

## Abstract

This paper represents new approaches for calculating, designing, and optimizing prestressed arched trusses with a tie member. Structural systems with long spans, such as trusses, beams, frames, etc., are subjected to a considerable/substantial risk of losing load-carrying capacity because of the different types of loads used. Some traditional design methods define the values of prestressing force in the tie member and internal forces in the truss elements to avoid this load capacity loss. However, the accuracy and limits of the determination of the forces are not necessarily known. The authors offer a new type of prestressed arched truss and some new approaches in the design and calculation process to solve these disadvantages. The study’s main objectives were to design an innovative and new geometric form of prestressed arched truss, which allows the development of high-value prestressing force, to optimize a new truss for reducing self-weight, increasing load-carrying capacity compared to its analogs. The force, stiffness matrix, and simulated annealing methods were used during the study. A new advance to the optimization of prestressed arched truss suggested by the authors reduces the self-weight and improves the load capacity of the truss by 8–17%, depending on the span.

## 1. Introduction

It is essential to use innovative and modern research methods in designing and calculating processes to satisfy load capacity, economic, disaster risk, and other demands submitted to the engineering structures. One of these methods is a prestressing technique of the structures. Generally, the prestressing technique’s principle involves introducing internal forces into the structure, which oppose those induced when external loads are applied. Structural systems with big sizes, such as trusses, beams, frames, arcs, or long supports, are subjected to a considerable risk of losing load-carrying capacity because of the different types of loads used. Introducing prestressed steel structures in the design process is among the most encouraging method to minimize the mass of deck or bridge systems [1].

Researchers are concerned with studying the load-bearing capacity of prestressed structural systems. The main benefit of using prestressing methods in a structural system is: (a) allows the use of cross-sections smaller, leading to less depletion of natural resources (aggregates, metal, coal, and others); (b) it reduces structural self-weight and hence small foundations, with immediate savings in construction; (c) reduces the costs of transport and assembly [2].

Prestressing of structures widens the elastic working range of the material. Thus, prestressed steel structures are widely used to disperse seismic energy in earthquake zones, decrease the structure’s mass, and raise the fatigue strength of the system. These considerations are especially relevant in long-spanning elements and high buildings, considering the challenging technological load [3,4].

**Figure 1:**
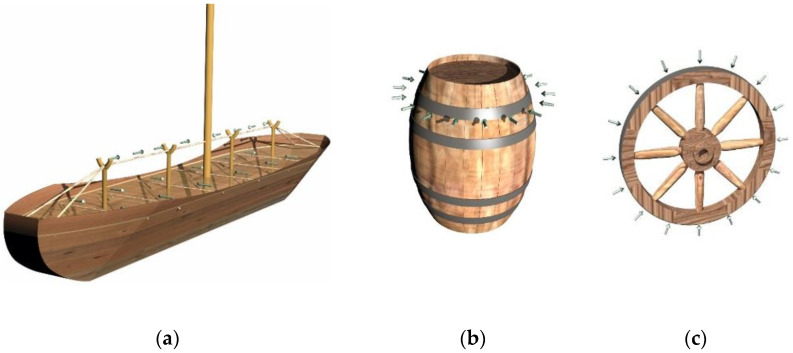
Application of the prestressing technique in different structures: (**a**) Ancient Egyptian prestressed ship; (**b**) prestressing used for barrels; (**c**) prestressed wheel.

Prestressing technology for structures was established many years ago, both for constructing new structures and for the rehabilitation of current ones. The use of prestressing in the structure of Egyptian sailing ships dates back to 2700 BC. They applied ropes and turnbuckles to keep the ship’s sides at the same level (Figure 1a). It was also used in everyday objects such as barrels, wheels, umbrellas, musical instruments, etc. (Figure 1b,c). While prestressing the wheel, iron rings were removed and tightly rolled to provide further rigidity and strength [5].

It was not until the mid-1800s that researchers began using prestressing techniques in bridge designs. In the United States, the trusses of Howe were patented in 1840, using wood material for the chord elements and cast-iron material for the diagonal and vertical ties. Dischinger and Magnel were the first to apply prestressing technology to steel structures. In the following years, many prestressed steel structures were built worldwide, especially in the USA, UK, and Germany, demonstrating that prestressed steel structures can provide structural and economic efficiency compared with non-prestressed existing systems [5].

In the literature [6], researchers investigated the use of prestressing cable as a tie member and steel as an element material in arched trusses to increase the load capacity and reduce weight. In the tested trusses, almost 40% of structural efficiency increased by adding the tie member (cable). The impact of the tensile and compressive parameters of cable-in-tube systems are studied in the literature [1], experimentally and numerically. The presence of cables enhanced the tensile ability of the elements, and at the same time, the action of prestressing forces extended the elastic range of the working material.

In the literature [5], the researchers examined the simply supported prestressed steel beams with tie members (tendons) and deviators. The primary outcome of this research was that because the buckling stress reduces, therefore, the prestressing force rate rises, which applies to the tendons, and consequently, the beam’s load-carrying capacity increases with the number of deviators.

Huang et al. [7] investigated the experimental behavior of prestressed concrete-filled steel tubes of truss girders. The experimental results demonstrated that the flexural stiffness and flexural strength of prestressed concrete-filled steel truss girders increase as the prestressing force magnitude develops.

In the studies [2,8], researchers offered an advanced prestressed steel girder structure for bridges with corrugated webs and two bottom tubes connected by trusses and without trusses. This advanced improvement in bridge design significantly reduces its self-weight and facilitates its construction.

A particular type of queen-post truss was studded numerically—via the finite element method and experimentally [9]. The system is lighter, easier to handle, robust and stiffer than a simple beam. Piyasena et al. [10] studied the blast resistance of cable trusses and cable net facades under blast loading. Gao et al. [11] invented unbonded prestressing tie members with self-centering connections using enhanced sliding cable elements to dissipate seismic energy in earthquakes.

In [12,13,14], an innovative approach is presented by which the load capacity and serviceability characteristics of cold-formed prestressed steel beams were improved using prestressing methods. Yang et al. [15] used polymer laminates reinforced with carbon fiber to strengthen the reinforced concrete structures. This method effectively reduced the cost of the system.

Yu et al. [16] presented new ways to optimize three-span prestressed columns to maximize the overall system efficiency.

Wang et al. [17] conducted optimization and buckling analysis of prestressed steel columns. The presented optimization approach accurately predicted the optimal cross-arm length related to the maximum critical buckling stresses. Wang et al. [4] discussed the analytical model of seismic behavior of prestressing bridge columns in earthquake zones, the numerical simulation and the experimental study executed in this research.

The literature [18,19] conducted an experimental study and numerical simulation of hollow-core slabs reinforced with fixed steel rods and prestressed steel tie members.

Segal et al. [20] conducted multi-objective optimization of suspended pedestrian bridge structures with steel and polyester rope as tie members. In the literature [13,21,22,23,24,25,26,27], optimization of concrete post-tensioned box girder bridges, road vaults and buttressed earth-retaining walls are provided. In the literature [28,29,30], durability, structural evaluation, and structural analysis conducted for carbon fiber-reinforced polymer (CFRP) strands and CFRP truss girders were considered.

The paper [31] allows evaluation of the prestressing method in Coventry cathedral. It describes how problems with the prestressing might manifest themselves and whether anything could be monitored to determine whether corrosion was occurring. In the literature [3], authors considered buckling analysis of columns out of the elastic region of the material. The behavior of prestressed trusses has also been explored [22,32,33,34,35,36,37]. Yepes-Bellver et al. [38] discussed surrogate modelling to optimize CO_2_ emissions for post-tensioned concrete slab bridge decks, reporting that a cost increase of less than 1% reduces CO_2_ emissions by more than 2%. In the literature [39,40,41,42,43], structural analyses, structural efficiency, and experimental study of prestressed truss systems were considered.

The main disadvantage of the before-considered design methods was that researchers did not always know the accuracy and limits of the definition of prestressing force value. In addition, there was a need for several approaches for sizing (definition of cross-sections) of truss elements, but obtained the best solution from the point of view of minimization of the self-weight of the truss was impossible. The next disadvantage was that drastically different unloading forces that arose after prestressing the arched truss (the first loading phase) in the top and bottom chords. It becomes necessary to increase cross-sections for the elements of the bottom chord; after loading the truss with exploitation loads (the second phase of loading), the analyses indicate that the bottom chord does not need as much cross-section as it was chosen in the prestressing phase. All these problems caused increases in the self-weight and cost of the truss.

**Figure 2:**
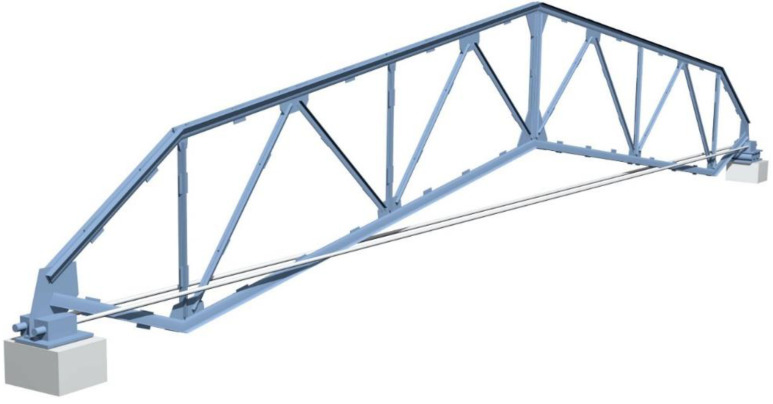
A new type of prestressed arched truss with a tie member offered by the authors.

The authors offered a new type of prestressed arched truss with a tie member (cable) to solve the above-mentioned disadvantages. They provided new approaches to the design and calculation process (Figure 2).

The main goals of the research were: (a) To design an innovative and new geometric form of prestressed arched truss, which allows the development of high-value prestressing force; (b) To solve the unloading and materials consumption exceeding problem of the bottom chord of the new truss by structural improvement; (c) To develop approaches in the calculation method that would allow specifying and raising of the magnitude of the prestressing force of a new truss beyond their “traditional theoretical” limit; (d) To optimize a new truss by the simulated annealing (SA) method for reducing self-weight, increasing load-carrying capacity, rigidity, reliability, and fatigue resistance compared to its analogues; (e) To extend the elastic region of working material of a new type of prestressed truss; (f) To simplify the calculation methods of statically indeterminate prestressed trusses, i.e., instead of several approaches for defining values of truss parameters (prestressing force in the cables, cross-sections of the truss elements), defining them with a particular single approach.

During the study, the force and stiffness matrix methods were used to define force values in the truss elements. The optimization of the arched truss was done using metaheuristic optimization, simulated annealing (SA) and gradient methods. The analytical method of Euler was applied in compression, which, on the other hand, was based on Hooke’s law. The prestressed arched truss was studied according to the equilibrium equations of mechanical theory.

The advantage of the performed method in determining the prestressing force of the truss when compared with traditional ones is that the prestressing force is derived analytically, allowing accurate reflection of real working data.

## 2. Problem Formulation

As mentioned above, prestressing is a structure in which initial stresses are created artificially, as opposed to the exploitation loads. The meaning and efficiency of the prestressing method are to extend the elastic working region of the material, raise the load-carrying capacity, and increase the rigidity and fatigue resistance of the prestressed structures compared with non-prestressed structures [1,5,6,44,45].

### 2.1. General Aspects of Understanding the Prestressing Method

**Figure 3:**
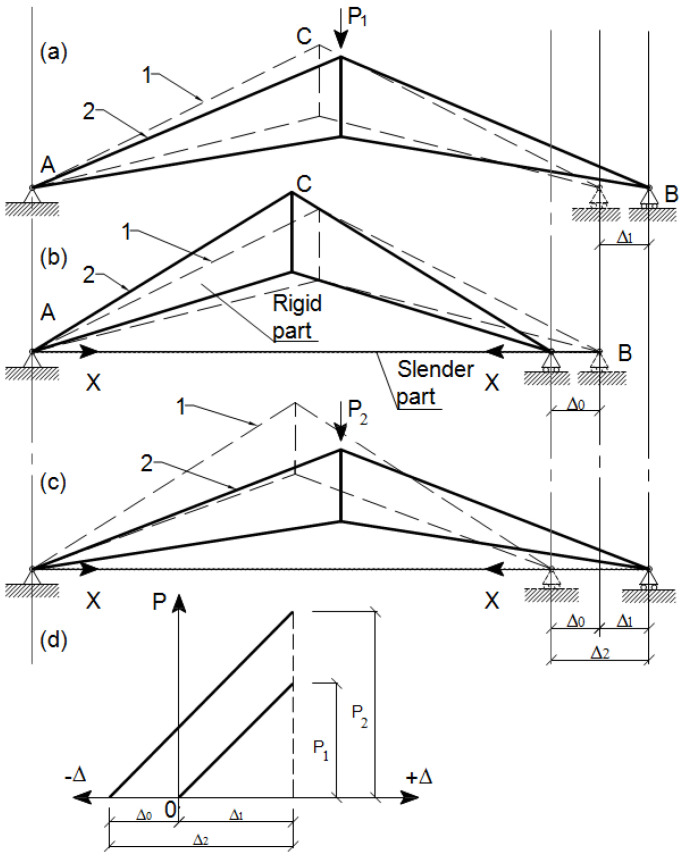
Hierarchy of the structural efficiency of the arched truss. (**a**) Non-prestressed truss; (**b**) prestressed truss; (**c**) loaded prestressed truss; 1—dotted line—initial position; 2—solid line—final position; (**d**) graph comparing the prestressed trusses’ parameters to the non-prestressed trusses’ parameters.

The simple truss presented in Figure 3 demonstrates the structural efficiency of the prestressing method, such as extending elastic range, rising load-carrying capacity, increasing rigidity and fatigue resistance, etc. Figure 3a depicts a non-prestressed arched simple truss in the initial state before loading (Figure 3a, position 1, dotted lines). It consists of a rigid part, i.e., the primary structure, without the tie member (slender part). After loading with a unit nodal force $P_{1}$ the support B deflects on the value $\Delta_{1}$ (Figure 3a, position 2, solid lines).

Figure 3b depicts a prestressed arched truss with a tie member. It consists of a rigid part, i.e., the primary structure, and a slender part, i.e., the tie member (high-strength cable). Dotted lines on the scheme indicate the initial state of the truss before prestressing (Figure 3b, position 1). At the first loading stage, the truss is prestressed by force (X). As a result, the support B deflects on the value $-\Delta_{0}$ (Figure 3b, position 2).

Figure 3c depicts a prestressed arched truss with a tie member. At the second loading stage, the truss is loaded with nodal force $P_{2}$. This force initially overcomes prestressed deformations ($-\Delta_{0}$), and after that, it starts to work as a standard truss with tie members. The support B deflects the value $\Delta_{2}=\Delta_{0}+\Delta_{1}$. The dotted lines indicate the initial position of the truss (Figure 3c, position 1).

When comparing some of the prestressed trusses’ parameters to the non-prestressed trusses’ parameters, the following result was obtained. From Figure 3c,d, it is evident that the elastic working region of the material was extended because $\Delta_{2}>\Delta_{1}$. Additionally, the load-carrying capacity of the truss was increased because $P_{2}>P_{1}$ (Figure 3d). This productive result is explained by the hierarchy of structural efficiency of the prestressed truss.

The simple arched steel truss with a tie member was studied as an example represented in Figure 3 studying the sturdy and buckling behavior of the prestressed simple arched steel truss is an appropriate step to understand and evaluate the reliability of more complex trusses [17].

### 2.2. Static Analysis of the Arched Trusses with a Cable

**Figure 4:**
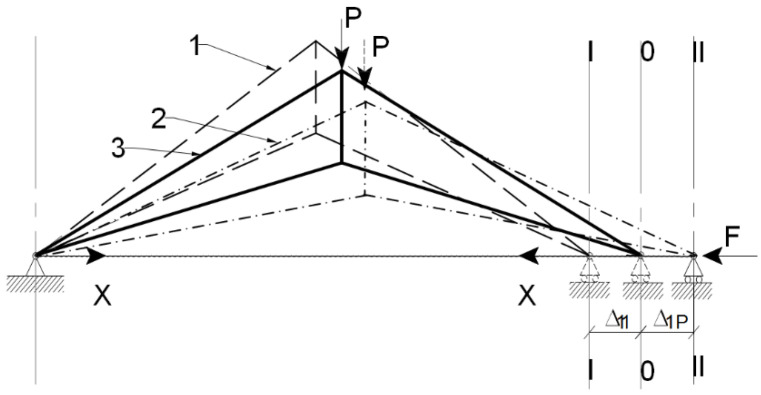
Prestressed arched truss with tie member. (1) Prestressing of the truss by force X at the I–I axis; (2) Loading of the truss by force P at II–II axis; (3) Restoring of equilibrium by some force at the 0–0 axis (neutral axis).

Figure 4 depicts the same prestressed arched truss with a tie member for static analysis. According to the check result, the truss is indeterminate to the first degree. Because the force in the tie member is determined, the tie member will be selected as the redundant. This means “cutting out” of that member so as not to maintain the force. As a result, the truss turns into a statically determinate and stable system. In this system, the principle of superposition is used.

Thus, this truss is a statically indeterminate system, and the force method is used for calculation. After prestressing, the truss deflects on the value $\Delta_{11}$ by force X from the axis-0−0 (Figure 4, position 1—dotted lines). After this, the truss is loaded by force P. At first, it overcomes prestressed deflection ($\Delta_{11}$), and at last, it deflects on the value $\Delta_{1p}$ from the axis 0−0 (Figure 4, position 2—dash-dotted lines).

Then, force F is applied to right-side support for restoring equilibrium (Figure 4, position 3, solid line), and this support is moved to the axis 0−0. The horizontal displacement ($\Delta_{1P}$) of the support due to the nodal load (P) and the horizontal displacement ($\Delta_{11}$) of the support due to an increase in horizontal force (X) in the tie member must equal zero [6,17].

Additionally, therefore, the following equation is obtained:

(1) $$\;\;\Delta_{11}+\Delta_{1p}=0$$

As it is well known from structural mechanics,

(2) $$\Delta_{11}=X_{1}\delta_{11}$$

Here, $X_{1}$ is the force arisen in the cable; $\delta_{11}$ is the deflection induced by a unit force; $\Delta_{1p}$ is the deflection induced by a live load.

Inputting $\Delta_{11}$ from (2) into (1), the following equation is derived:

(3) $$X_{1}\delta_{11}+\Delta_{1p}=0$$

From Equation (3), the force arisen in the cable can be determined as horizontal force,

(4) $$X_{1}=-\frac{\Delta_{1p}}{\delta_{11}}$$

Adoption of Maxwell’s formula for arched trusses with a tie member gives the following value of coefficients:

(5) $$\delta_{11}=\overset{i=n}{\underset{i=1}{\sum }}\frac{N_{1i}^{2}l_{i}}{E_{i}A_{i}}+\frac{l_{c}}{A_{c}\cdot E_{c}}$$

and,

(6) $$\Delta_{1p}=\overset{i=n}{\underset{i=1}{\sum }}\frac{N_{pi}N_{1i}l_{i}}{E_{i}A_{i}}$$

where $N_{pi}$ is the strain (force) caused by the nodal load impact on the i-bar; $N_{1i}$ is the force risen in the truss elements in the cable from the unit strain; $l_{i}$ is the design length of the i-bar of the truss; $E_{i}$ and $E_{c}$ are the elasticity moduli of the bar and cable; $A_{i}$ and $A_{c}$ are the cross-section areas of the i-bar and cable of the truss; and $l_{c}$ is the span (design length) of the cable.

The first member of Equations (5) and (6) refers to the primary structure (arched truss), and the second member refers to the tie member (cable). The force along the cable is known and is equal to 1 ($N_{1}=1$). Because the stiffness of the cable ($A_{c}\cdot E_{c}$) at tensioning and the length ($l_{c}$) of the cable have constant values, they can be moved out of the sum (or integral) of Formula (5). Inputting Equations (5) and (6) into Equation (4) gives the possibility to derive the formula for the calculation of self-stressing force:

(7) $$X_{1}=\frac{\sum_{i=1}^{i=n}\frac{N_{pi}N_{1i}l_{i}}{E_{i}A_{i}}}{\sum_{i=1}^{i=n}\frac{N_{1i}^{2}l_{i}}{E_{i}A_{i}}+\frac{l_{c}}{A_{c}\cdot E_{c}}}$$

Additionally, the force in the cable is:

(8) $$N_{c}=X+X_{1}$$

The force value in the cable is composed of prestressing and self-stressing forces. For the first approach in determining the magnitude of the prestressing force, designers assign (set) the value of the prestressing force in the cable:

(9) $$N_{c}\approx 0.6\cdot N_{bc}$$

where, $N_{bc}$ is the force value in the bottom chord of the statically determinate truss (primary structure) caused by the exploitation load (nodal load). This is defined beforehand. After this, from Equation (8), it is possible to define the force value within the cable:

(10) $$X=N_{c}-X_{1}$$

After calculating the unknown reaction force (X), the force acting in any rod of the truss is performed by the following formula:

(11) $$N_{i}=N_{pi}+N_{1i}\cdot X$$

In Equation (11), the superposition principle is used [1,2].

### 2.3. Algorithm for the Calculation of Prestressed Trusses

Here, is a proposed algorithm of the calculation of prestressed trusses by the force method: (a) generating the model geometry of the truss; (b) defining the boundary conditions of the truss (restraining the nodes); (c) defining the material properties of the elements of the truss using standard types of sections; (d) applying loads on the truss; (e) generating a DCF table (design combination of forces); (f) static analysis of the primary truss (defining the values of the forces within the rods of the truss without prestressing); (g) selecting sections of the rods in the first approximation without prestressing; (h) assigning the prestressing force within the cable: $N_{c}\approx 0.6\cdot N_{\mathrm{bc}}$; (i) defining the expected forces within the elements of the bottom chord of the truss acting out of cable $N_{c}$; (j) preventing the buckling risk of compressed rods: $N_{\left(1-H\right)}=N_{p\left(\mathrm{bc}\right)}+N_{1\left(\mathrm{bc}\right)}\cdot N_{c}$; (k) selecting the section of the rods of the bottom chord of the truss: $A=N/\left(\varphi R_{y}\right);$ (l) defining the value of the forces within the cable: $N_{c}=\left(N_{p}-R_{y}\cdot A\right)/N_{1;}$ (m) defining the value of forces within the truss elements: $N_{i}=N_{pi}+N_{1i}\cdot N_{c}$; (*n*) selecting the sections of the rods by $N_{i};$ (o) defining the section of the cable by the formula: $A_{c}=N_{c}/\left(0.65\cdot R_{y}\right)$; (p) selecting the section of cable (diameter, area of section…) by formula $A_{c}$ using standard types of sections; (q) defining the self-stressing force by Equation (7); (r) defining the prestressing force of the cable: $X=N_{c}-X_{1};$ (s) defining the value of forces within the truss elements: $N_{i}=N_{pi}+N_{1i}\cdot X$ (second approach); (t) selecting the sections of the truss by $N_{i}$ (second approach).

### 2.4. Disadvantages in the Calculation Method

The above-mentioned algorithm of calculation—the force method is entirely suitable for the analysis of trusses that are statically indeterminate to the first degree. Despite this, it has some disadvantages, and it is in the iterative nature of the structural design. It is well known that defining the force values and sizing of elements of statically indeterminate trusses is a classic chicken-and-egg problem: acting forces in the primary structure (for sizing truss elements) cannot be defined until the force value within the tie member (cable) is known (for sizing cable). On the other hand, the force value within the tie member (cable) cannot be known until the acting forces in the primary structure (truss) have been defined. To escape the paralysis of this situation, an assumption has to be made about the force value within the cable and through an iterative process, approach a final solution.

For this reason, the authors have to make several iterations. Using this approach may reach satisfactory results. However, these results will not be the best solution, as high accuracy and precision of the cross-section of the truss elements cannot always reached. In this connection, a problem occurs during the iteration of prestressing forces that will later have an essential impact on the entire process of truss optimization, i.e., on achieving the minimal weight of the truss. One of the objectives of this study is to find the value of prestressing force in the tie member that finally allows for reaching a minimum weight of the truss [6,8,17].

### 2.5. Disadvantages in the Structural Design Process of the Arched Truss with Tie Members (Cable)

The choice of magnitude of the prestressing forces is one of the main points in the structural design process because it is strictly related to the cost of the truss [5,46,47,48]. The more the prestressing force is, the lower the cross-section is needed for sizing the truss elements. The authors considered two main disadvantages of the arched truss.

**Figure 5:**
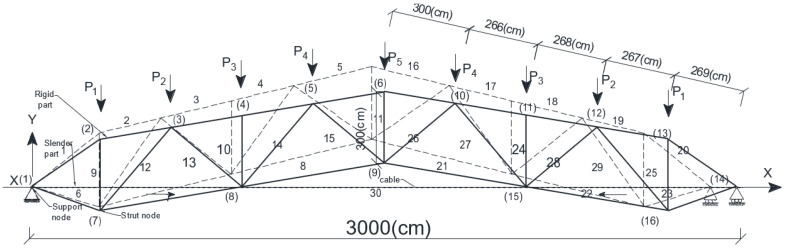
A new type of prestressed arched truss with a cable (tie member).

The first disadvantage: after tensioning (the first phase of loading) of the truss by cables (Figure 2, Figure 3b and Figure 5) in the top and bottom chords, drastically different unloading forces arise—the bottom chord is compressed (−), while the top chord is tensioned (+). The location of the tie member causes the reason for these differences. Thus, the further the tie member is located from the truss’s neutral axis, the less prestressing force arises in the primary structure. In most existing prestressed arched trusses, the tie member is located between the strut nodes (Figure 2 and Figure 5). Therefore, if the tie member is located further from the neutral axis, it does not allow for the possibility of developing the highest values of prestressing forces [5].

The second disadvantage: after prestressing the truss by a horizontal force (the first phase of loading), it is necessary to size the bottom chord caused by compressive forces and the top chord caused by tensile forces, see Table 1 and Table 2. Sizing the bottom chord by these forces results in larger cross-sections in the bottom chord than would be necessary during the exploitation phase of loading by a nodal (vertical) force.

After loading the truss with a nodal (vertical) force (the second phase of loading), it is necessary to size the bottom chord caused by tensile forces and the top chord caused by compressive forces, see Table 1 and Table 2. Sizing the bottom chord by these forces results in fewer cross-sections in the bottom chord than sized during the prestressed phase of loading (by a horizontal force).

This unloading is because buckling failure occurs at a lower force in compression than in tension. This can be explained from the hierarchy of structural efficiency of the strained elements: tensioned members tend to be more (maybe several times) efficient than compressed members. For this reason, there arises unloading elements, and this causes material consumption in the bottom chord of the truss [1].

## 3. Problem Solution

### 3.1. Geometric, Physical and Mechanical Characteristics of a New Type of Prestressed Arched Truss

To solve the problems mentioned above, the authors designed a new prestressed arched steel truss with a tie member. Figure 5 depicts the design scheme of the new truss with tie members, and as it was mentioned in Figure 2, it is presented in a 3D model. This research analyses the behavior of a new type of truss having a medium span as a roofing element for structures. Specifically, the impact on the truss’ response, the value of prestressing force within the cable, the rate of nodal loads on the truss, and changing the span of the truss are thoroughly investigated by the authors [5].

In designing the new type of arched truss (Figure 5), the case is considered when the span equals l=30 m (subsequent checks were made for spans: 24 m, 36 m, 42 m). The truss consists of a rigid part (primary structure—statically determined truss) and a slender part (tie member—cable).

**Figure 6:**
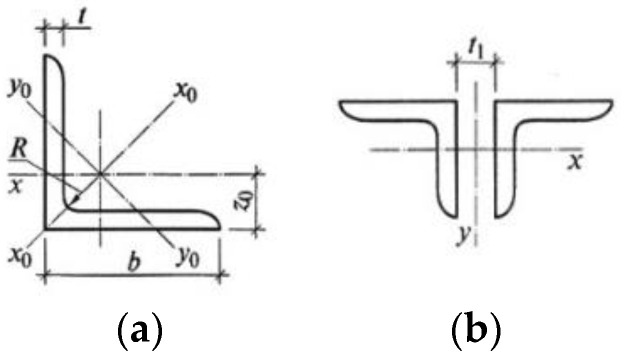
The view of the cross-section of the equal-leg angles, back-to-back type: (**a**) equal-leg angle; (**b**) composed section.

The primary structure is an arched truss and represents a rod system. It has an upper chord (1–5, 16–20), a lower chord (6–7–8, 21–22–23), a lattice—vertical and diagonal elements (9–15, 24–29), support nodes (1, 14), strut nodes (7, 16) and loading nodes (2–6, 16–20). The slender part is a tie member—cable (30). The cross-sections of the truss’s upper, lower, vertical and diagonal elements are made out of equal-leg angles. Geometric dimensions of the cross-section of the truss elements are presented in Table 1 and Figure 6.

As a material for the primary structure (rigid part) of the truss, a low-carbon hot-rolled mild steel was used (S245), and for the slender part (tie member—cable), high-strength steel was used. Table 2 presents the material properties of the prestressing cable. Design resistance of the angular truss elements was: $R_{y}=24$ (kN/cm^2^); elastic modulus (Young’s modulus) of the angular truss elements was: E=21,000 (kN/cm^2^); design resistance of the cable was: $R_{u}=200$ (kN/cm^2^); and the elastic modulus (Young's modulus) of the cable was: E=16,000 (kN/cm^2^).

### 3.2. Problem Solution in the Structural Design Process Method

The new structure of the truss (Figure 2 and Figure 5) provides avoidance of the above-stated drawbacks.

Problem solution of the first disadvantage—as mentioned above, after tensioning the truss by the cable (Figure 2 and Figure 3b) in the top and bottom chords, drastically different unloading forces arise, and it limits the development of the high-value prestressing forces. To solve the problem mentioned above, the location of the tie member needs to change and move towards the neutral axis of the truss as close as possible. Specifically, it needs to be moved from the strut nodes to the truss’s support nodes (Figure 2 and Figure 5). This improvement is based on the calculation and analyses of the new truss. It can be concluded that the improvement gives the possibility to reach maximal and very similar magnitudes of the stresses in the upper and lower chords of the truss. This improvement enables the effective use of the materials.

Problem solution of the second disadvantage—a material consumption exceeds the bottom chord of the truss arises. After moving a tie member of the truss very close to the neutral axis of the truss from the strut nodes to the support node (Figure 5), an opportunity arises to overcome the second drawback. When the tie member is moved to the support node, the bottom chord no longer becomes as stressed (compressed) compared to when the tie member was located at the strut nodes, consequently, the bottom chord does not need as much cross-sectional area. This improvement also allows for higher values of the prestressing force in the tie member. Thus, this action causes a reduction in material consumption in the truss.

### 3.3. Problem Solution in the Calculation Method

The offered new type of arched truss prestressed with a tie member (cable) is a statically indeterminate system (Figure 5). Presented above was an algorithm of the prestressed arched truss using the force method (Section 2.4), where an unknown (redundant) force was assigned to the cable. In this case, using the force method in the calculation of such types of trusses does not give an easy, convenient way for obtaining the best solutions, i.e., the possibility for obtaining precise and accurate values of the prestressing force in the cable and, consequently, force values in the truss elements. These values are necessary for minimizing the truss elements’ cross-sectional areas (weight). However, the value of the prestressing force in the cable is chosen at the designer’s discretion, which is not always exact.

The authors used the matrix stiffness method instead of the force method to determine the force values in the truss elements and the tie member. Apart from this, the sizing process of the cross-sections of the truss elements was conducted using the metaheuristic optimization method—simulated annealing—in the cable and the truss elements instead of the iteration mentioned in the above approach (Section 2.4).

The weight of the truss, as well as the force in the cable, were the considered variables. In the calculating process of the truss, the computer program “MATLAB” was used.

### 3.4. Calculation of the New Model of the Prestressed Arched Truss Using the Matrix Stiffness Method and a Numerical Example

To obtained the force value in the 2D planar truss elements, the authors calculated it using the matrix stiffness method.

Next, the data were used to obtain numerical values: the model of the 2D truss, its boundary conditions, and the load case are presented in Figure 5. Cross-sections for the truss elements are presented in Table 1 and Table 2. Nodal load—dead weight of elements of the model: $P_{1}=57.6;\;P_{2}=57.6;\;P_{3}=83.4;\;P_{4}=91.8;\;$kN. Nodal load—live load: $P_{1}=85.4;\;P_{2}=85.4;\;P_{3}=73;\;P_{4}=90.7;\;$kN.

Model geometry of the truss was generated (Figure 2 and Figure 5) using coordinates given in Table 3, and node connections are given in Table 4. The number of truss nodes was 16, and the number of truss elements was 30 and are shown in Table 4.

Two degrees of freedom were considered at every node: horizontal and vertical displacements. For every truss bar, its (4 × 4) element stiffness matrix was defined by using Equation (12) to (14).

The stiffness matrix of the element was defined in global coordinates with K. Note that c and s are the cosine and sine directions for the elements, so the stiffness matrix was obtained in global coordinates. Structural stiffness matrix $K_{s}$ was defined using the procedures of matrix assembly. Restrained degrees of freedom were eliminated to consider bearing conditions: u_x_(1), u_y_(1) and u_y_(14).

(12) $$L=\sqrt{{\left(x_{2}-x_{1}\right)}^{2}+{\left(y_{2}-y_{1}\right)}^{2}}$$

Additionally,

(13) $$c={\left(x_{2}-x_{1}\right)}^{2}/L\;s={\left(y_{2}-y_{1}\right)}^{2}/L$$

Additionally,

(14) $$K=\frac{EA}{L}\left[\begin{array}{cccc} c^{2} & cs & -c^{2} & -cs\\ cs & s^{2} & -cs & -s^{2}\\ -c^{2} & -cs & c^{2} & cs\\ -cs & -s^{2} & cs & s^{2} \end{array}\right]$$

Then, the forces P acting at each loaded node with the force vector P were determined as follows: the authors found the components of each applied force in the directions of the global degrees of freedom. The vector of force was assembled by placing these components of force in the force vector in the right place.

Deflections d were found by inverting the stiffness matrix and multiplying it by the load vector:

(15) $$d=K_{s}/p$$

*d* is the structural deflection vector in the global coordinate system; p is the structural load vector in the global coordinate system; $K_{s}$ is the structural stiffness matrix in the global coordinate system and $p=K_{s}d$ [9,21,22].

Multiplying these deflections by the stiffness matrix of every element, internal bar forces T were computed. As the value of force of each bar is known, the reactions can be obtained from the balance equations.

All truss elements can be checked, obtaining compressions or tension stresses from the internal forces and cross-sectional areas, including the cable.

### 3.5. Optimization of Prestressed Truss

A simulated annealing (SA) algorithm was used to optimize the structural characteristics/parameters. It is a heuristic optimization technique that has been successfully used in numerous conditional optimization problems, particularly in structural optimization, as it has the advantage of solving problems independently for the discrete or continuous formulation of variables. SA is a local improvement algorithm with strategic acceptance of solutions.

The application of the SA algorithm to the problem discussed here has been done in the following way: The starting point is the initial solution of the problem, that is, a truss that would be a feasible solution to the problem. This solution is slightly altered in successive iterations by an operator, which randomly modifies the cross-section of one or more truss rods or the prestressing cable of the truss. In each iteration, the resulting solution, obtained by applying this operator, replaces the previous one and is also a solution to the problem if it is lighter than the previous one. The feasibility is given by compliance/accordance with Section 3.4 above. However, sometimes more expensive solutions than their predecessor will also be accepted. The decision to accept these worse solutions is made at random, but with a probability given by the expression:

(16) $$P=e^{-\frac{\Delta W}{T}}$$

where, ΔW is the weight increment, and T is temperature, which decreases during the process. When the algorithm is unable to improve the prevailing/current lattice, after a certain number of attempts, the algorithm stops, indicating that the local optimum has been reached.

The acceptance of such decisions, which worsens the result of the algorithm, prevents early convergence into a low-quality optimum. The probability of accepting worse solutions is reduced by reducing the parameter T guaranteeing the convergence to an optimum since with T=0 the probability of accepting a worse solution (probability of making worse decisions) is zero.

Kirkpatrick proposed an algorithm described in this way. It is based on the analogy with the Metropolis models for crystal formation in melted metals at high temperatures when they are cooled. The position of the atoms within the crystal structure tends to have the lowest/minimum energy states. Metropolis uses Boltzman’s formula to give atoms a certain probability of worsening the state of energy and thus represents the movement of atoms as they do in the actual process of solidification/hardening. Hence, the parameter T is traditionally referred to as “temperature” [21,22].

Used algorithm. In this case, the optimization task has 16 variables, all representing the cross-section of the rods of the truss, plus the cross-section of the prestressing cable. The possible cross-sections are taken from Table 1 and Table 2 and correspond to the hot-rolled equal-leg angles (back-to-back) profile type, and steel cables.

**Figure 7:**
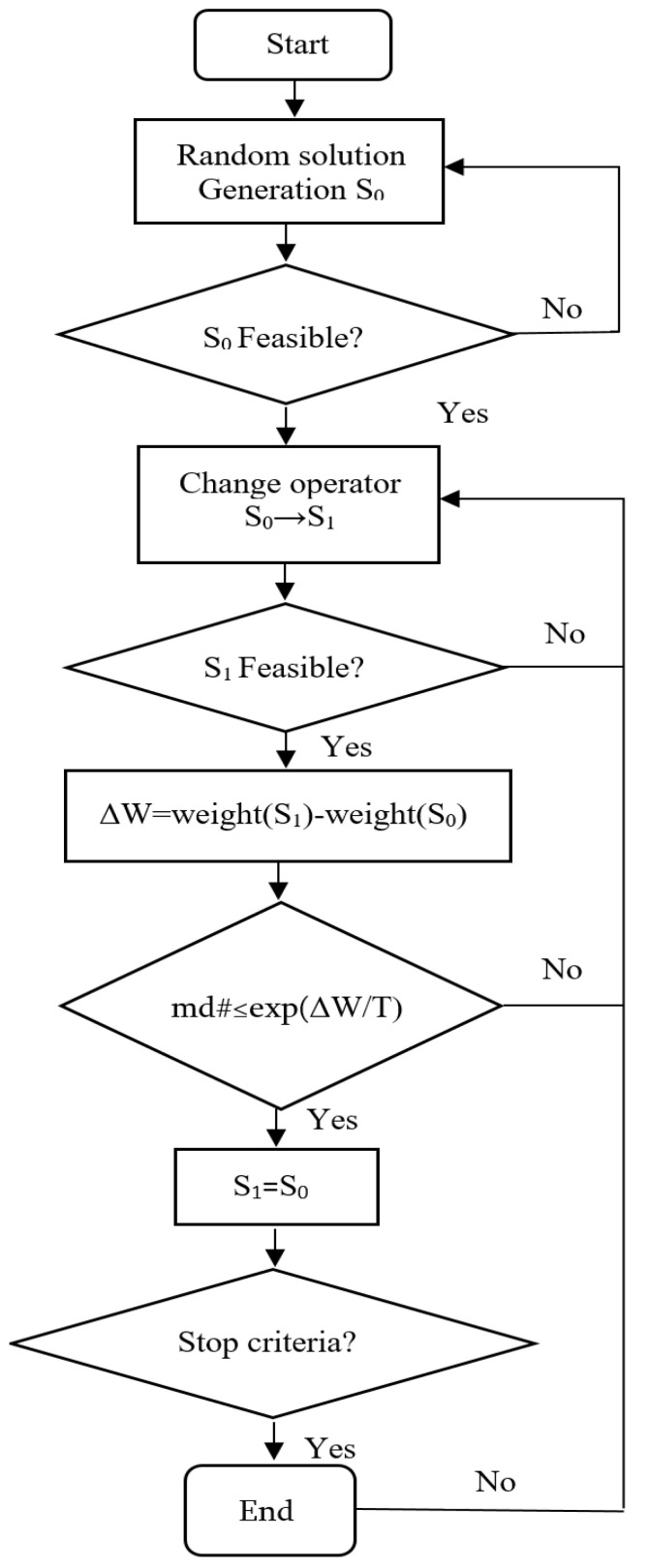
Flow chart of the optimization of the prestressed arched truss.

The designed algorithm follows the diagram presented in Figure 7. It automatically generates lattices of the truss, for which it selects one of the profiles for each bar (cross-section) from the possible ones from Table 1 and cable sections from Table 2. This selection is random. If this solution is not feasible, it starts again until a solution appears. At that time point, it evaluates the weight, and that solution is termed the initial one. From that moment, the iterative process begins, as described in the previous section. The starting temperature is taken as a percentage of the weight of the initial solution so that the value is scaled to the size of the structure.

The shift operator that changes the solution $S_{0}$ to $S_{1}$ randomly selects the predetermined number of rods also randomly decides whether to increase or decrease the cross-section of that rod by one step, always according to the series of cross-sections from Table 1. If the cable is randomly selected, it is altered similarly, increasing or decreasing the cross-section in a step according to Table 2.

There is a mathematical demonstration of the convergence of the SA algorithm to a global optimum due to the assimilation of the algorithm’s behavior to the mathematical conditions under which this demonstration was made. The SA is usually programmed using a constant temperature for a finite number of iterations, after which it is reduced geometrically by multiplying it by a coefficient less than one. This coefficient is known as the cooling coefficient.

The number of iterations in which the temperature remains constant fulfills/meets the conditions of the Markov model (the probability that a given result will occur in a chain of stochastic events depends only on the previous result), so this series of iterations is a Markov chain. The temperature decreases during the process following a geometric law. After applying each Markov chain, a cooling coefficient that is lower than the unit is multiplied by the previous chain’s temperature.

In the application of the algorithm for the problem here, calibration tests have been carried out to find out the appropriate parameterization of the algorithm, arriving at the establishment of the following parameters of the algorithm: Markov chain lengths = 500, initial temperature 5% of the weight of the initial solution and cooling coefficient = 0.9. The shift operator modifies the 5-rod cross-section in each iteration. As a criterion for stopping the algorithm, it is accepted that an optimum is reached and the optimization process is exhausted when no improvements are achieved in a complete Markov chain.

Since the algorithm is randomized at several points, the results achieved in each execution vary. For this reason, each case is executed nine times to obtained an average result representative of the algorithm’s behavior. The result shown in this paper for each execution is the one with the lowest weight of those nine. The typical execution time of the algorithm varies between 118 and 230 s on a computer with an Intel^®^ Core ™ i7 processor at 3.33 GHz.

Application of the algorithm. The SA algorithm just described was used to optimize the weight of the prestressed arched truss with cable of Figure 5. The truss was subjected to the following loads: 10 kN, 15 kN, 20 kN, 25 kN.

**Figure 8:**
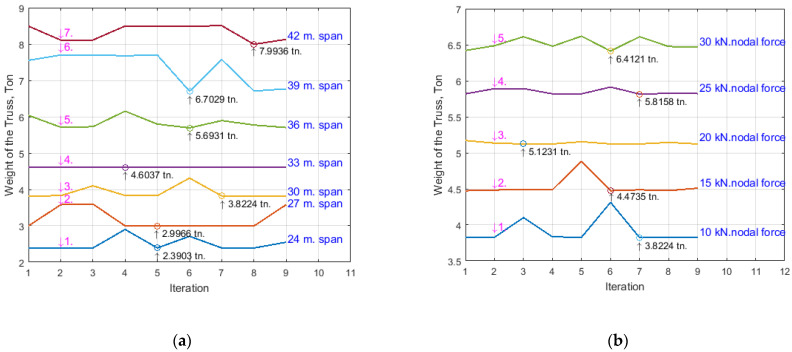
Optimization of the weight of the prestressed arched truss. (**a**) Optimization of the weight (in tons) of the prestressed arched truss at different spans; (**b**) Optimization of the weight (in tons) of the prestressed arched truss at different nodal forces.

These trusses were optimized for spans of 24, 27, 30, 33, 36, 39, and 42 m, obtaining the weights in Table 5 and Table 6 below (see Figure 8).

### 3.6. Sizing of the Cross-Section of the Truss Elements

The sizing of the cross-sections of the axially tensile and axially compressed elements of the truss, when optimized by the SA method, was carried out according to the following expressions:

For tensile elements:

(17) $$\frac{N}{A}\leq R_{y}$$

For compressed elements:

(18) $$\frac{N}{A}\leq \varphi R_{y}$$

where, N is the internal axial force aroused in the cross-section of the truss element, $R_{y}$ is the steel yielding limit, A is the cross-section area, and φ is the longitudinal bending coefficient (reduction factor for the relevant buckling mode) that is calculated according to the bar slenderness and steel yielding limit.

## 4. Results and Analyses

The optimization results of the weight of the prestressed arched truss with various iterations at different spans are presented in Table 5. Here, $P_{0}$ is the prestressing force; the symbol “Itr” means iteration; the symbol “time” means optimization time; symbols “24 m”, “27 m”, “30 m”, “33 m”, “36 m”, “39 m”, and “42 m” are the spans (meter) of the truss; and the nodal force is 10 kN.

The optimization results of the weight of the prestressed arched truss with various iterations at different nodal forces are presented in Table 6. Here, 10, 15, 20, 25, and 30 kN are nodal forces of the truss; and the span of the truss is 30 m.

A diagram depicting the optimization results of the weight of the prestressed arched truss at different spans is plotted in Figure 8a, accordingly to Table 5. A diagram depicting the optimization results of the weight of the prestressed arched truss at different nodal forces is plotted in Figure 8b, accordingly to Table 6. The main goal of the research was to obtained an arched truss with minimal weight at different spans and different nodal forces.

Figure 8a shows the optimization results of the new truss at a nodal force of 10 kN. Curves 1–7 indicate the weight optimization of the trusses with spans 24, 27, 30, 33, 36, 39, and 42 m. Consequently, the minimum weights were 2.3903; 2.9966; 4.6037; 5.6931; 5.8224; 6.7029; and 7.9936 tons, respectively.

Figure 8b shows the new truss’s optimization results at a span of 30 m. Curves 1–5 indicate the weight optimization of the trusses with nodal forces 10, 15, 20, 25, and 30 kN. Consequently, the minimum weights were 3.8224; 4.3735; 5.1231; 5.8158; and 6.4121 tons, respectively.

**Figure 9:**
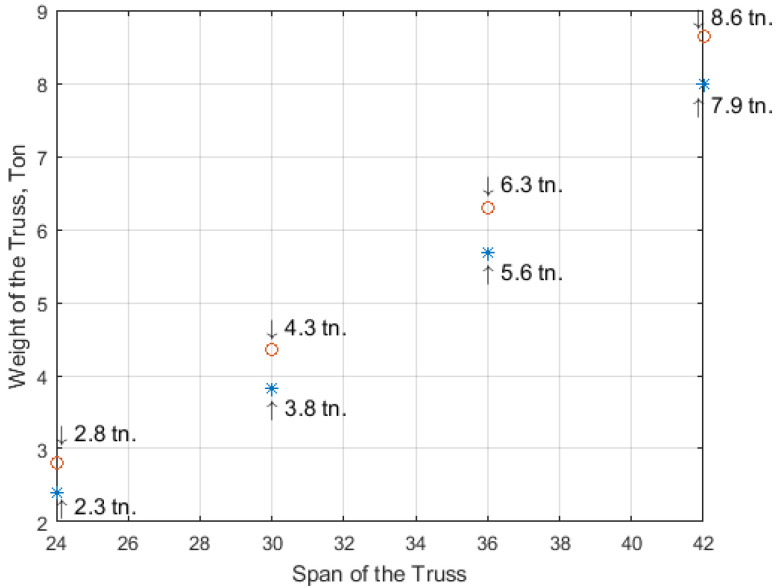
Comparative analysis of prestressed arched trusses calculated with the classical (ο) and optimized (*) methods.

Figure 9 depicts the comparative analyses of the calculation of the prestressed arched truss by the traditional approach (force method) and the stiffness matrix after SA optimization.

The rings in Figure 9 denote the results of the truss calculation using the classical (force) method, and the results obtained by SA are denoted with asterisks.

The truss spans were 24 m, 30 m, 36 m, and 42 m. The nodal load was 10 kN. The results confirm the new approach’s advantages and accuracy in truss design and calculation methods.

Based on the theoretical and numerical analyses, the following results were obtained:

(1) A new geometric and innovative form was discerned for a prestressed arched truss that allows the development of higher values of prestressing force;
(2) A new geometric and innovative truss was discerned that does not have unloading elements in the bottom chord, and the materials consumption exceeding problem was solved;
(3) A new approach to the calculation (metaheuristic optimization) of a prestressed arched truss has been developed, resulting in the determination of the highest values of prestressing force with high accuracy in the tie member.
(4) A new type of prestressed arched truss was optimized, as a result the minimal weight of the truss in different spans (Figure 8a) and at different loads (Figure 8b) was obtained;
(5) By the new approaches offered by the authors, the weight of the truss was reduced, and the load capacity, rigidity, reliability, and fatigue resistance of the new truss were increased (see Figure 9);
(6) The rate of prestressing force was increased (the asterisks are below the rings in Figure 9), as a result the elastic region of the working material was extended.

## 5. Conclusions

- It is recommended to use this new type of prestressed arched truss with a tie member as an innovative long-span roof system optimized and designed by the authors;
- It is recommended to adopt the innovative technique proposed in the process of designing the geometric form of a new type of prestressed arched truss. Thus, the tie member of the truss should be moved from the strut node towards the support node (Figure 2 and Figure 5), to eliminate unloaded elements in the truss structure and to develop a high rate of prestressing force in the primary structure of the truss;
- This research recommends using the performed approach in calculating prestressed arched trusses, where the highest values of the prestressing force are determined by the metaheuristic optimization and matrix stiffness methods;
- The research in this paper recommends the new approaches to obtain a truss with the lowest gravity, as the location of points of minimal weight of the prestressed arched truss by metaheuristic optimization were established and presented in Figure 8a,b;
- The optimization of the prestressed arched truss reduces the self-weight and increases the load capacity of the truss by 8–17%, depending on the span (see Figure 9);
- The new approach to increasing the rate of prestressing force of the truss achieves elastic region of working material expansion by almost 12–14%;
- It is recommended to use the new approaches, instead of several approaches for defining values of truss parameters (prestressing force in the cables, cross-sections in the truss elements), as far as it is possible to define them with a particular single approach;
- Reducing the weight of the new truss consequently reduces CO_2_ emissions.
- To obtain the prestressed arched truss with minimum weight is necessary to conduct future research not only to optimize the cross-sectional area, but also the truss topology (shape formation). In particular, it will be very relevant to optimize the shape of the chords, the boom lift, the type of lattice, and the number of panels of prestressed trusses.

## Figures and Tables

**Table 1:** Geometric dimensions of the equal-leg angles (back-to-back) by GOST 8509–93 (shortened) of the trusses and by Figure 6.

b, Width of a Cross-Section, mm	t, Thickness, mm	A, Area of a Cross-Section, cm^2^	$i_{x}$, Radius of Gyration, cm	$i_{y}$, Radius of Gyration, cm	m, Mass per Meter, kg/m
50	5	4.88	1.53	2.45	3.77
63	5	6.13	1.94	2.96	4.81
70	5	6.86	2.16	3.22	3.38
75	6	8.78	2.30	3.44	6.89
80	6	9.38	2.47	3.65	7.36
90	6	10.6	2.78	4.03	8.33
90	7	12.3	2.77	4.06	9.64
100	7	13.8	3.08	4.44	10.8
100	8	15.6	3.07	4.47	12.2
110	8	17.2	3.39	4.87	13.5
125	8	19.7	3.87	5.46	15.5
125	9	22.0	3.86	5.48	17.3
140	9	24.7	4.34	6.09	19.4
140	10	27.3	4.33	6.11	21.5
160	10	31.4	4.96	6.91	24.7
160	11	34.4	4.95	6.93	27.0
160	16	49.1	4.89	7.03	38.5
180	11	38.8	5.60	7.74	30.5
180	12	42.2	5.59	7.76	33.1
200	12	47.1	6.22	8.55	37.0
200	14	54.6	6.20	8.60	42.8
200	16	62.0	6.17	8.64	48.7
220	16	68.6	6.81	9.42	53.8
250	16	78.4	7.76	10.6	61.5
250	20	97.0	7.71	10.7	76.1

**Table 2:** Steel cable’s geometric, physical and mechanical dimensions by GOST 3068-66 (shortened) for the tie member (cable) of the truss.

Diameter of the Cable, cm	Cross-Section Area, cm^2^	Mass of the Cable (1 m), kg	Breaking Force of the Cable, kN
3.8	6.61	5.86	907.5
4.2	8.15	7.24	1120
4.6	9.87	8.75	1350
5.1	11.74	10.45	1610
5.5	13.81	12.25	1895
5.9	16.01	14.20	2200
6.3	18.37	16.30	2525
6.8	20.90	18.55	2825

**Table 3:** Number and position of nodes.

Joints	X	Y	Joints	X	Y
1	0	0	9	15	1.5
2	3	2.1	10	18	3.9
3	6	2.7	11	21	3.3
4	9	3.3	12	24	2.7
5	12	3.9	13	27	2.1
6	15	4.5	14	30	0
7	3	−0.9	15	21	0.3
8	9	0.3	16	27	−0.9

**Table 4:** Number and position of nodes.

Bars	Joint (i)	Joint (j)	Bars	Joint (i)	Joint (j)
1	1	2	16	6	10
2	2	3	17	10	11
3	3	4	18	11	12
4	4	5	19	12	13
5	5	6	20	13	14
6	1	7	21	9	15
7	7	8	22	15	16
8	8	9	23	16	14
9	7	2	24	15	11
10	8	4	25	16	13
11	9	6	26	9	10
12	7	3	27	10	15
13	3	8	28	15	12
14	8	5	29	12	16
15	5	9	30	1	14

**Table 5:** Optimization results of the weights of the new truss at different spans.

	**Span—24 m**			**Span—27 m**			**Span—30 m**			**Span—33 m**		
**Itr.**	**Weight**	**P_0_**	**Time**	**Weight**	**P_0_**	**Time**	**Weight**	**P_0_**	**Time**	**Weight**	**P_0_**	**Time**
1	2.3903	200	118.553	2.3903	200	118.553	2.3903	200	118.553	2.3903	200	118.553
2	2.3903	200	220.806	2.3903	200	220.806	2.3903	200	220.806	2.3903	200	220.806
3	2.3903	200	108.674	2.3903	200	108.674	2.3903	200	108.674	2.3903	200	108.674
4	3.0042	890	117.505	3.0042	890	117.505	3.0042	890	117.505	3.0042	890	117.505
5	2.3903	200	167.809	2.3903	200	167.809	2.3903	200	167.809	2.3903	200	167.809
6	2.7126	570	241.135	2.7126	570	241.135	2.7126	570	241.135	2.7126	570	241.135
7	2.3903	200	220.899	2.3903	200	220.899	2.3903	200	220.899	2.3903	200	220.899
8	2.3903	200	206.569	2.3903	200	206.569	2.3903	200	206.569	2.3903	200	206.569
9	2.5488	380	146.296	2.5488	380	146.296	2.5488	380	146.296	2.5488	380	146.296
	**Span—36 m**			**Span—39 m**			**Span—42 m**			**Span—45 m**		
**Itr.**	**Weight**	**P_0_**	**Time**	**Weight**	**P_0_**	**Time**	**Weight**	**P_0_**	**Time**	**Weight**	**P_0_**	**Time**
1	2.3903	200	118.553	2.3903	200	118.553	2.3903	200	118.553	-	-	-
2	2.3903	200	220.806	2.3903	200	220.806	2.3903	200	220.806	-	-	-
3	2.3903	200	108.674	2.3903	200	108.674	2.3903	200	108.674	-	-	-
4	3.0042	890	117.505	3.0042	890	117.505	3.0042	890	117.505	-	-	-
5	2.3903	200	167.809	2.3903	200	167.809	2.3903	200	167.809	-	-	-
6	2.7126	570	241.135	2.7126	570	241.135	2.7126	570	241.135	-	-	-
7	2.3903	200	220.899	2.3903	200	220.899	2.3903	200	220.899	-	-	-
8	2.3903	200	206.569	2.3903	200	206.569	2.3903	200	206.569	-	-	-
9	2.5488	380	146.296	2.5488	380	146.296	2.5488	380	146.296	-	-	-

Itr.—Iterations; Weight—(Ton); P_0_—Prestessing Force (kN); time—(second); Spans—24 m–42 m. Nodal force—10 kN.

**Table 6:** Optimization results of the weights of the new truss at different nodal forces.

	Nodal Force—15 kN			Nodal Force—20 kN			Nodal Force—25 kN			Nodal Force—30 kN		
Itr.	Weight	P_0_	Time	Weight	P_0_	Time	Weight	P_0_	Time	Weight	P_0_	Time
1	4.4736	280	238.436	5.1709	440	118.123	5.8186	650	113	6.4201	660	-
2	4.4832	270	187.765	5.1351	380	198.251	5.8907	440	-	6.4864	650	-
3	4.4928	280	270.759	5.1231	400	184.268	5.8917	430	-	6.6117	870	-
4	4.4892	280	247.101	5.1231	400	167.945	5.8186	630	-	6.4793	630	-
5	4.8831	640	196.089	5.1542	430	152.751	5.8186	630	124	6.6192	870	-
6	4.4736	270	174.967	5.1231	400	224.255	5.9131	370	164	6.4121	660	-
7	4.4867	280	238.909	5.1231	400	204.561	5.8158	620	116	6.6117	870	-
8	4.4747	280	190.498	5.1446	370	125.209	5.8248	640	115	6.4756	610	-
9	4.5094	320	163.796	5.1231	400	198.452	5.8214	630	132	6.4675	610	-

Itr—Iterations; Weight—(Ton); P_0_—Prestessing Force (kN); time—(seconds); Span of the truss—30 m.

## Data Availability

The data used to support the findings of this study are available from the corresponding author upon request.
